# Catheter ablation vs. drug therapy in the treatment of atrial fibrillation patients with heart failure: An update meta-analysis for randomized controlled trials

**DOI:** 10.3389/fcvm.2023.1103567

**Published:** 2023-03-08

**Authors:** Chun Lin, Mingyan Sun, Youbin Liu, Yongkang Su, Xiao Liang, Shouyuan Ma, Ping Zhu, Yuming Fu, Jianfeng Liu

**Affiliations:** ^1^ Department of Cardiology, The Second Medical Center and National Clinical Research Center for Geriatric Diseases, Chinese People’s Liberation Army General Hospital, Beijing, China; ^2^ Department of General Medicine and Geriatrics, Shenzhen Qianhai Shekou Free Trade Zone Hospital, Shenzhen, China; ^3^ Department of Ninth Health, The Second Medical Center and National Clinical Research Center for Geriatric Diseases, Chinese PLA General Hospital, Beijing, China; ^4^ Department of Cardiology, The Second Affiliated Hospital of Hainan Medical University, Haikou, China; ^5^ Department of Geriatrics, The Second Medical Centre, Chinese PLA General Hospital, Beijing, China; ^6^ Key Laboratory for Biomechanics and Mechanobiology of the Ministry of Education, Beijing Advanced Innovation Centre for Biomedical Engineering, School of Biological Science and Medical Engineering, Beihang University, Beijing, China

**Keywords:** atrial fibrillation, heart failure, catheter ablation, drug therapy, randomised controlled trials, mortality

## Abstract

**Background:**

Atrial fibrillation (AF) and heart failure (HF) often coexist. The treatment of AF in patients with HF has been challenging because of the ongoing debate about the merits of catheter ablation vs. drug therapy.

**Methods:**

The Cochrane Library, PubMed, and www.clinicaltrials.gov were searched until June 14, 2022. Inclusion criteria were catheter ablation compared with drug therapy in adults with AF and HF in randomized controlled trials (RCTs). Primary outcomes consisted of all-cause mortality, re-hospitalization, change in left ventricular ejection fraction (LVEF), and AF recurrence. Secondary outcomes referred to quality of life [QoL; measured by the Minnesota Living with Heart Failure Questionnaire (MLHFQ)], six-minute walk distance (6MWD), and adverse events. The PROSPERO registration ID was CRD42022344208.

**Findings:**

In total, nine RCTs with 2,100 patients met the inclusion criteria, with 1,062 for catheter ablation and 1,038 for medication. According to the meta-analysis, catheter ablation significantly reduced all-cause mortality compared with drug therapy [9.2% vs. 14.1%, OR: 0.62, (95% CI: 0.47–0.82), *P *= 0.0007, *I*^2^*^ ^*= 0%], improved LVEF [MD: 5.65%, (95% CI: 3.32–7.98), *P < *0.00001, *I*^2^*^ ^*= 86%], reduced AF recurrence [41.6% vs. 61.9%, OR: 0.23, (95% CI: 0.11–0.48), *P < *0.0001, *I*^2^*^ ^*= 82%], decreased the MLHFQ score [MD: −6.38, (95% CI: −11.09 to −1.67), *P *= 0.008, *I^2 ^*= 64%] and increased 6MWD [MD: 17.55, (95% CI: 15.77–19.33), *P < *0.0001, *I*^2^*^ ^*= 37%]. Catheter ablation did not increase the re-hospitalization [30.4% vs. 35.5%, OR: 0.68, (95% CI: 0.42–1.10), *P *= 0.12, *I*^2^*^ ^*= 73%] and adverse events [31.5% vs. 30.9%, OR: 1.06, (95% CI: 0.83–1.35), *P *= 0.66, *I*^2^*^ ^*= 48%].

**Interpretation:**

In AF patients with HF, catheter ablation improves exercise tolerance, QoL, and LVEF and significantly reduced all-cause mortality and AF recurrence. Although the differences were not statistically significant, the study found lower re-hospitalization and approximate adverse events with improved catheter ablation tendency.

**PROSPERO registration ID:**

CRD42022344208.

## Introduction

AF and HF often coexist. HF premises AF with an incidence of 10%–50%, depending on its severity (1–5). AF can also aggravate HF and increase mortality (6). Antiarrhythmic drugs have limited potential to maintain sinus rhythm. Significant side effects, including arrhythmia and increased mortality risk, limit drug therapy for AF in patients with HF (7–9). Moreover, the benefits of sinus rhythm may be offset by the side effects of drugs (10–12). It's worth noting that long-term sinus rhythm maintenance with minimal side effects has been the focus of discussion. Several studies (13–16) have demonstrated that while catheter ablation was more effective than medications in maintaining sinus rhythm, serious adverse events were still a concern. The results of earlier RCTs (17–21) indicated that catheter ablation caused more adverse events than drug treatment in patients with AF and HF. According to recent RCTs (22, 23), catheter ablation had fewer adverse outcomes than drug therapy. In that way, catheter ablation would no longer be controversial when the side effects were less than those of medication. Thus, based on these inconsistent research conclusions for several endpoints, our meta-analysis compared the effects of catheter ablation with drug therapy for patients with AF and HF using RCTs (17–25).

## Methods

### Search strategy and selection criteria

PRISMA (26) (Preferred Reporting Items for Systematic Reviews and Meta-analyses) statement was followed for this study. We searched PubMed, The Cochrane Library, and www.clinicaltrials.gov databases until 14 June 2022. A PICO (patient, intervention, comparison, and outcome) approach was used to determine inclusion criteria: (1) Randomized controlled trials with clinical outcomes had been published. (2) Patient: Persistent or symptomatic paroxysmal atrial fibrillation with NYHA ≥ II of chronic heart failure in adults (≥18 years). (3) The Intervention group: rhythm control using catheter ablation. (4) The Control group: pharmacotherapy. (5) Outcome: cardiovascular endpoints. Keywords used in the literature search included: “atrial fibrillation”, “heart failure”, and “catheter ablation”. The literature search was performed using keywords: [“atrial fibrillation” and “heart failure” and “catheter ablation”]. A manual review of the reference lists of relevant literature was conducted to determine which studies should be included.The primary outcomes consisted of all-cause mortality, re-hospitalization, change in LVEF and AF recurrence. Secondary outcomes included QoL, assessed by MLHFQ, 6MWD, and adverse events.

### Data analysis

Data were collected regarding the clinical trials' characteristics, patients’ baseline characteristics, intervention, and follow-up. The Cochrane Risk of Bias Tool (27) was used to evaluate the quality of individual studies. Intention-to-treat analysis was used in all comparisons. A two-tailed 95% confidence interval (CI) and odds ratios (OR) were calculated for categorical variables. A mean difference (MD) was calculated for continuous variables based on the mean and standard deviation. Cochran's *Q* test (28) was used to detect heterogeneity among eligible studies. For all comparisons, we calculated OR and MD estimates using a random-effects model (29). Heterogeneity analyses (Baujat plots, influence diagnostics, overall effect, and *I*^2^ heterogeneity) were used to test the consistency of the primary result. The publication bias was measured using Egger's test (30). Statistical significance was set at 0.05 for all *P*-values. Review Manager, version 5.3 (Copenhagen, Nordic Cochrane Centre, Cochrane Collaboration), Stata software, version 17.0 (Stata Corp., College Station, TX, United States), and R software, version 4.2.1, were used for statistical analysis.

## Results

### Overview of trials

**Figure 1:**
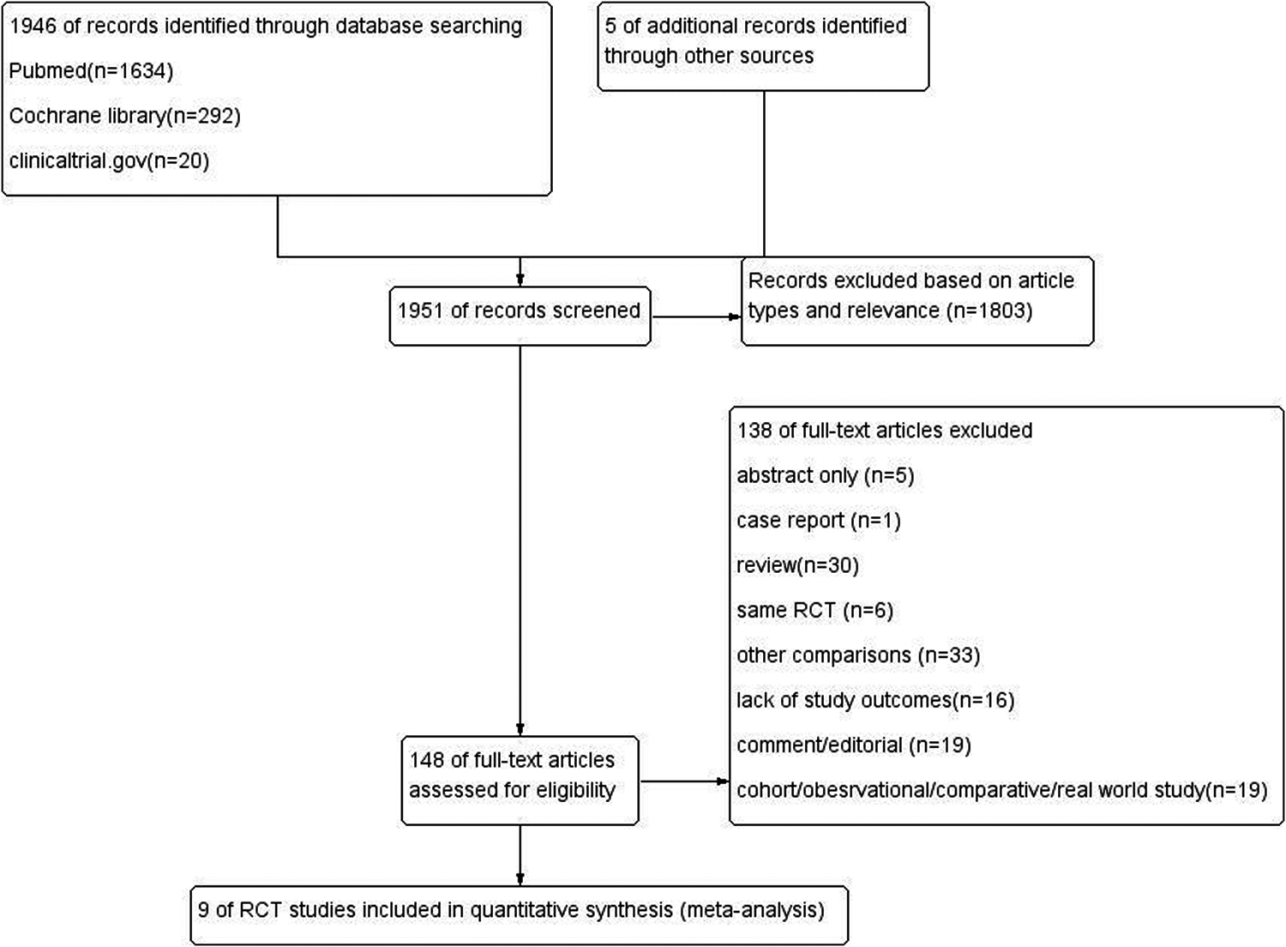
PRISMA Flow Diagram for study selection. RCT: randomized controlled trial. The Cochrane Library, PubMed, and www.clinicaltrials.gov databases are available until June 14, 2022.

Based on the preliminary screening of 1,951 references, 1,803 citations were eliminated due to article types and relevance. An additional 138 articles were excluded based on their titles and abstracts. Finally, 9 RCTs were included (*n* = 2,100). Among them, 1,062 cases were randomly divided into the catheter ablation group, and 1,038 cases were randomly divided into the drug therapy group (Figure 1). The characteristics of RCTs were shown in Table 1. Trials were judged to have a low risk of selection, detection, attrition, reporting, and other biases but a high risk of performance bias due to open-label designs (Supplementary Figures S3, S4). The risks of reporting bias were considered low for all-cause mortality, 6MWD, and adverse events, while bias existed for re-hospitalization, change in LVEF, AF recurrence, and QoL outcomes.

**Table 1:** Characteristics of RCTs.

Characteristic	MacDonald 2011	ARC-HF 2013	CAMTAF 2014	AATAC 2016	CAMERA-MRI 2020	CASTLE-AF 2018	AMICA 2019	CABANA HF sub-study 2021	RAFT-AF 2022
Sample size	41	52	50	203	62	363	140	778	411
Catheter ablation	22	26	26	102	47	179	68	378	214
Drug therapy	19	26	24	101	15	184	72	400	197
Mean age (SE), y									
Catheter ablation	62.3 ± 6.7	64 ± 10	55 ± 12	62 ± 10	60.5 ± 10.7	64	65 ± 8	68	65.9 ± 8.6
Drug therapy	64.4 ± 8.3	62 ± 9	60 ± 10	60 ± 11	65.5 ± 7.2	64	65 ± 8	67	67.5 ± 8.0
Male, *n* (%)									
Catheter ablation	17 (77)	21 (81)	25(96)	77 (75)	42 (89.3)	156(87)	60 (88)	207(55)	157 (73.4)
Drug therapy	15 (79)	24 (92)	23(96)	74 (73)	14 (93.3)	155(84)	66 (92)	226(57)	148 (75.1)
AF pattern	persistent AF	persistent AF	persistent AF	persistent AF	persistent AF	Parox-AF/Persis-AF	persistent/longstanding persistent AF	Parox-AF/Non-parox-AF	Parox-AF/Non-parox-AF
AF duration (months)									
Catheter ablation	44 ± 36.5	23 ± 22	24	8.6 ± 3.2	29 ± 30	NR	NR	13.2	14.5
Drug therapy	64 ± 47.6	24 ± 29	24	8.4 ± 4.1	24 ± 20	NR	NR	14.4	15
Randomization	Ablation vs. medical rate control	Ablation vs. medical rate control	Ablation vs. medical rate control	Ablation vs. amiodarone	Ablation vs. medical rate control	Ablation vs. medical rhythm and rate control	Ablation vs. best medical control	Ablation vs. medical rhythm and rate control	ablation-based rhythmcontrol VS medical rate control
Monitoring method	24-h Holter	48-h Holter, CIED	48-h Holter	CIED	ILR, dual chamber pacemaker or defibrillator	ICD or CRT-D	ICD or CRT-D	CABANA monitoring system or local recording devices	12-lead electrocardiogram
Primary end point	Change in LVEF	Change in VO2max	Change in LVEF	AF recurrence	Change in LVEF	All-cause death or HF hospitalization	the absolute increase in LVEF	Composite death, disabling stroke, serious bleeding, or cardiac arrest	All-cause mortality or heart failure event
Total follow-up, months	6	12	6–12	24	48	60	12	60	24

ARC-HF, a randomized trial to assess catheter ablation versus rate control in the management of persistent atrial fibrillation in heart failure; CAMTAF, a randomized controlled trial of catheter ablation versus medical treatment of atrial fibrillation in heart failure; AATAC, ablation vs. amiodarone for treatment of persistent atrial fibrillation in patients with congestive heart failure and an implanted device; CAMERA-MRI, catheter ablation versus medication in atrial fibrillation and systolic dysfunction; CASTLE-AF, catheter ablation for atrial fibrillation with heart failure; AMICA, catheter ablation versus best medical therapy in patients with persistent atrial fibrillation and congestive heart failure; CABANA HF sub-study, ablation versus drug therapy for atrial fibrillation in heart failure; RAFT-AF, randomized ablation-based rhythm-control versus rate-control trial in patients with heart failure and atrial fibrillation; AF, atrial fibrillation; CHD, coronary heart disease; CVD, cardiovascular disease; TIA, transient ischemic attacks; mo, month; ACEI, angiotensin-converting enzyme inhibitors; ARB, angiotensin receptor blockers; LVEF, left ventricular ejection fraction; QoL, quality of life; MLHFQ, minnesota living with heart failure questionnaire; NT-proBNP, n-terminal pro-brain natriuretic peptide; BNP, brain natriuretic peptide; NR, not report; CIED, cardiovascular implantable electronic devices; ILR, implantable loop recorder; ICD, implantable cardioverter-defibrillator; CRT-D, cardiac resynchronization therapy defibrillator; PVI, pulmonary vein isolation; HF, heart failure; NA, not available.

### Primary outcomes

#### All-cause mortality

**Figure 2:**
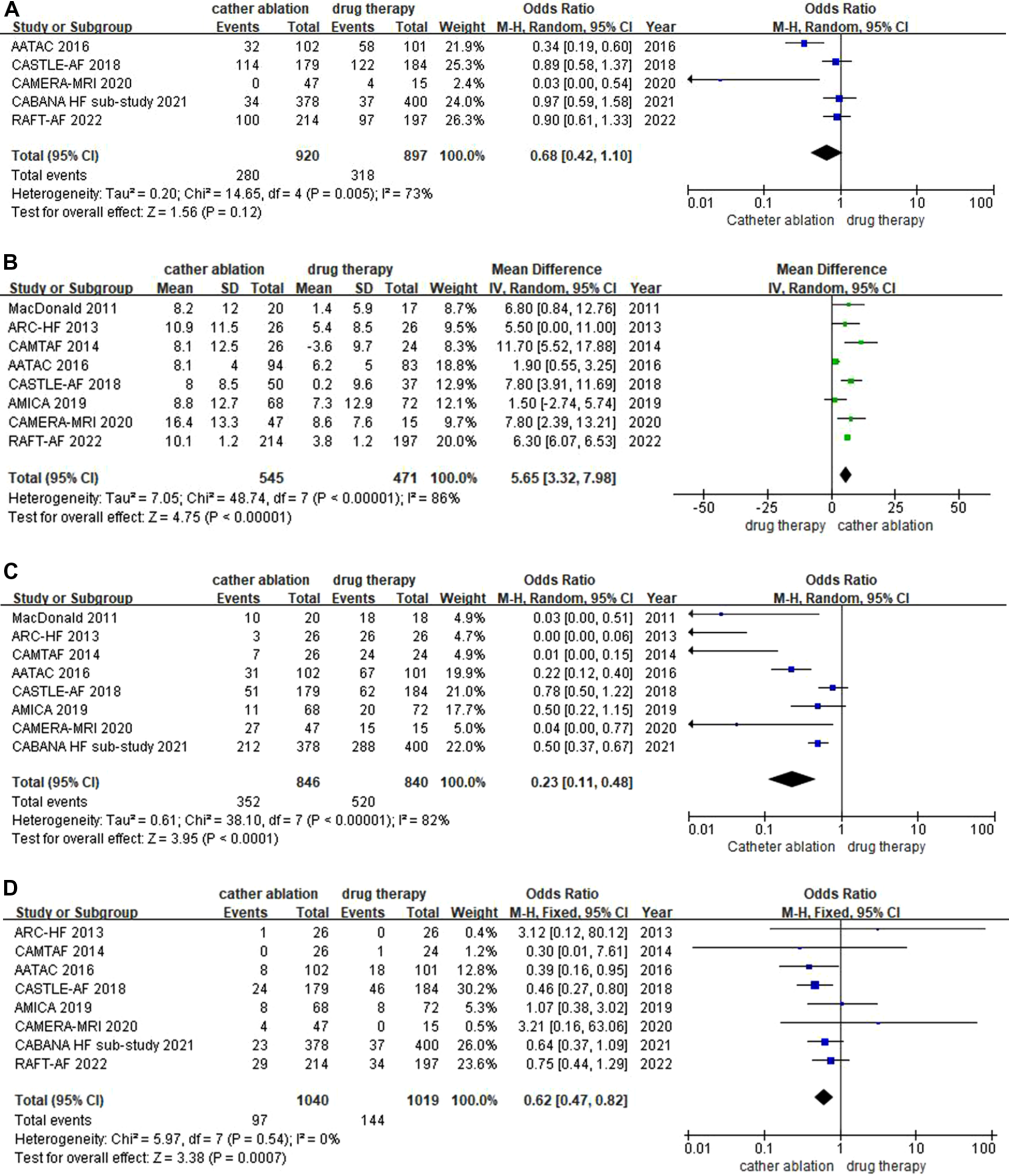
(**A**) Forest plot of Primary Outcomes-Composite of all-cause mortality. (**B**) Forest plot of Primary Outcomes-Re-hospitalization. (**C**) Forest plot of Primary Outcomes-Change in LVEF. (**D**) Forest plot of Primary Outcomes-AF recurrence.

A total of eight trials reported outcomes for all-cause mortality. A significant reduction in all-cause mortality was associated with catheter ablation [9.2% vs. 14.1%, OR: 0.62, (95% CI: 0.47–0.82), *P *= 0.0007, *I^2 ^*= 0%]. Heterogeneity analysis suggested that the included results were homogenous. The *P*-value indicated that the statistic is statistically significant (Figure 2A).

#### Re-hospitalization

There were five trials that provided data on re-hospitalizations. Catheter ablation did not increase re-hospitalization rates compared with drug therapy [30.4% vs. 35.5%, OR: 0.68, (95% CI: 0.42–1.10), *P *= 0.12, *I^2 ^*= 73%]. The heterogeneity of the combined effect size was high, and the results were not statistically significant (Figure 2B).

#### Change in LVEF

Changes in LVEF were assessed in 8 trials. The catheter ablation group had a greater increase in LVEF than the drug therapy group [MD: 5.65%, (95% CI: 3.32–7.98), *P < *0.00001, *I^2 ^*= 86%]. Despite the high heterogeneity, all studies showed improved LVEF in the catheter ablation group. Furthermore, the overall effect size was statistically significant when combined with the *P*-value (Figure 2C).

#### AF recurrence

There were 8 trials with AF recurrence that showed a lower rate of AF recurrence in the catheter ablation group [41.6% vs. 61.9%, OR: 0.23, (95% CI: 0.11–0.48), *P < *0.0001, *I^2 ^*= 82%]. Despite the high heterogeneity, fewer AF recurrence events were observed in each study, which, combined with the *P*-value, suggested that the overall effect size was statistically significant (Figure 2D).

### Secondary outcomes

#### Qol

**Figure 3:**
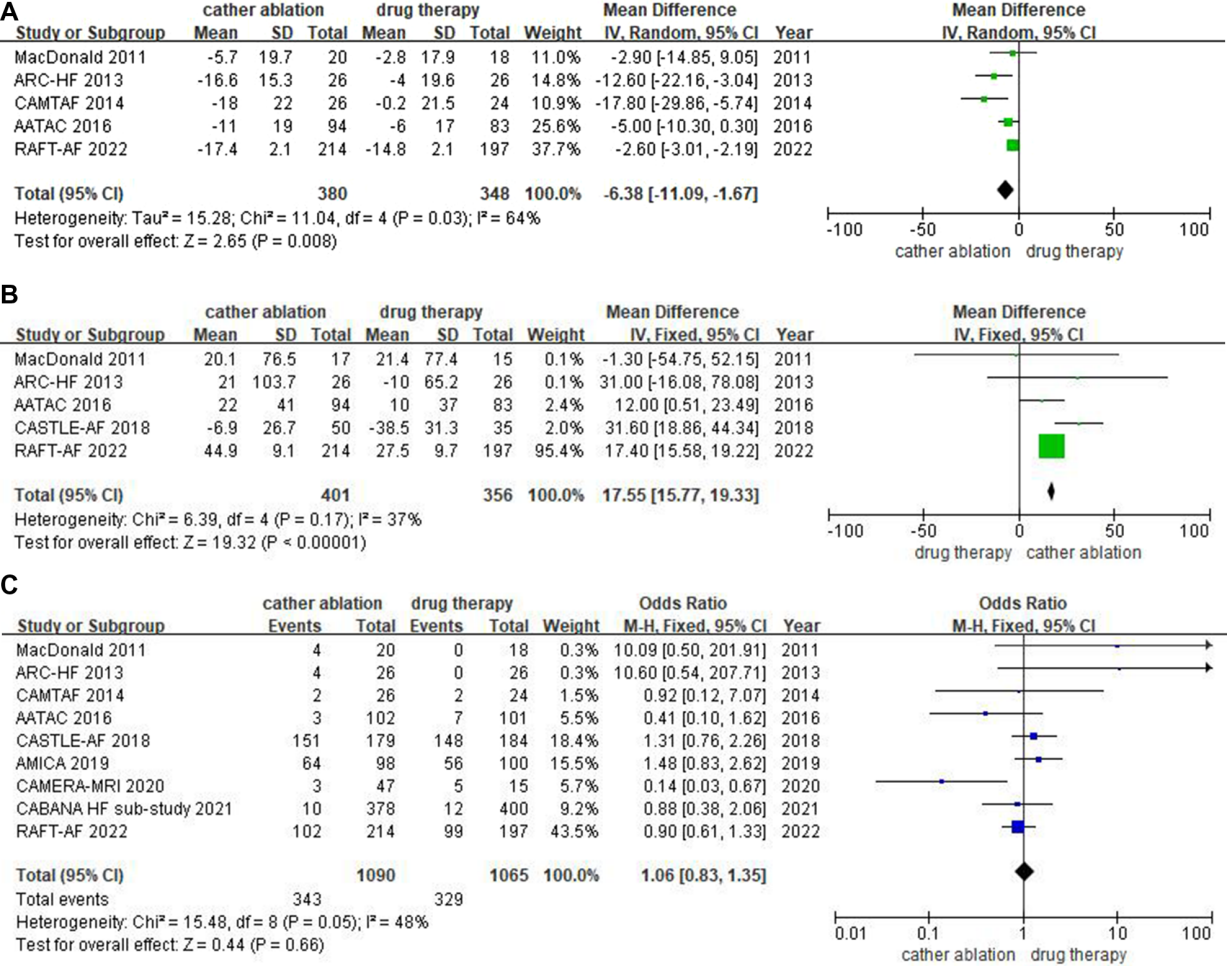
(**A**) Forest plot of Secondary Outcomes-Quality of life, (MLHFQ). (**B**) Forest plot of Secondary Outcomes-Distance on the 6-minute walk test. (**C**) Forest plot of Secondary Outcomes-Adverse events.

Five trials reported the outcome of QoL based on the Minnesota Living with Heart Failure Questionnaire (MLHFQ), which showed MLHFQ scores decreased significantly in catheter ablation groups [MD: −6.38 points, (95% CI: −11.09 to −1.67 points), *P *= 0.008, *I^2 ^*= 64%]. Despite the high heterogeneity, better MLHFQ scores were observed in all studies with the catheter ablation group when combined with the *P*-value. The overall size of effect was statistically significant (Figure 3A).

#### 6-minute walk distance (6MWD)

Data on 6MWD were available from five trials. Catheter ablation significantly increased 6MWD [MD: 17.55 m, (95% CI: 15.77–19.33 m), *P < *0.0001, *I^2 ^*= 37%]. Heterogeneity analysis suggested that the enrolled results were homogenous. The *P*-value indicates that the statistic is statistically significant (Figure 3B).

#### Adverse events

All 9 trials with adverse events showed that the rate of adverse events associated with catheter ablation was similar to that associated with medical control [31.5% vs. 30.9%, OR: 1.06 (95% CI: 0.83–1.35), *P *= 0.66, *I^2 ^*= 48%]. Heterogeneity analysis suggested that the homogeneity of the enrolled studies was good. The *P*-value indicated that the effect size was not statistically significant (Figure 3C).

#### Heterogeneity analysis

According to the heterogeneity analyses in Supplementary Figure S1, studies included in this review showed substantial heterogeneity. The regression-based Egger's test in Supplementary Table S1 showed no publication bias except for re-hospitalization and AF recurrence. Through trim-and-fill analysis (Supplementary Figure S2), for re-hospitalization, the estimated value of the combined effect size did not change significantly, which indicated that publication bias had little influence and the result was relatively robust. For AF recurrence, 3 studies were estimated to be theoretically missing. Based on 11 observed and imputed studies, the overall OR dropped from 8.038 (based on 8 observed studies) to 2.737 (95% CI: 0.491–15.254). The literature report on AF recurrence might therefore be more significant than it would be without publication bias. According to the funnel plot (Supplementary Figure S2), almost all imputed studies had a *P*-value above 10%. The publication bias might further explain the small-study effect. Further research may reveal that there is no statistically significant difference in AF recurrence between catheter ablation and drug therapy.

## Discussion

The treatment of AF in patients with HF has been challenging because of the ongoing debate about the merits of catheter ablation vs. drug therapy. The core issue is rhythm control vs. rate control. In 2020, the East-AFNET4 trial (31) showed that early rhythm control significantly improved the prognosis of AF patients. The EAST-AFNET4 trial established the dominant role of rhythm control strategies in atrial fibrillation. The rapid development of catheter ablation techniques has made it a mainstream approach to rhythm control. STOP-AF study (32) and EARLY-AF study (33) showed that catheter ablation was significantly superior to drug therapy in reducing the incidence of atrial fibrillation at the treatment endpoint. However, Whether catheter ablation is superior to drug therapy in patients with AF and HF remains to be observed. This meta-analysis showed that catheter ablation of AF patients with HF significantly reduced all-cause mortality, improved LVEF, decreased AF recurrence, lowered the MLHFQ score, and increased the 6MWD. Distinctively, re-hospitalization and adverse events with improved tendency were not statistically different between the catheter ablation and drug therapy groups. According to these results, catheter ablation is remarkably more effective than drug therapy for controlling the cardiac rhythm.

Although previous meta-analyses (34, 35) also reported similar benefits, they were all based on early RCTs with small samples and more serious adverse events in the ablation groups. Researchers and physicians worldwide have been interested in whether the adverse events of catheter ablation have improved in recent years. Updated meta-analyses are inevitable with the release of more extended follow-up studies of existing RCTs (20, 22) and new large-scale RCT results (23) in recent years. In our meta-analysis, catheter ablation could reduce all-cause mortality by 38% compared with medical therapy. This might be attributed to the decreased AF recurrence and improved LVEF. Our meta-analysis also showed that catheter ablation could reduce the incidence of AF recurrence by 77%. There was a high risk of early recurrence of AF, and the burden of AF might not be reduced within 3–6 months of study initiation, especially when repeat ablation was required. The previous study (36) had observed regression of left atrial dilatation after ablation and suggested that this might help reduce the recurrence of arrhythmias. In our meta-analysis, catheter ablation improved LVEF by 5.65% over medical therapy. Improvement in cardiac function is an important indicator. The majority of studies (17–20, 23–25) indicated that catheter ablation improved cardiac function in AF and HF patients. However, the AMICA study (21) showed no statistical difference in changes in LVEF in the ablation group. In the CASTLE-AF study (19), catheter ablation's benefits were observed most after 12 months following the procedure. Since the AMICA study follow-up ended after 12 months, a longer follow-up might be necessary for patients with severe heart failure. It was possible that catheter ablation to improve ventricular function and clinical benefits would take longer than expected. The delayed therapeutic effect may be due to the remodeling of the left ventricle over time. One explanation for the insufficient increase in LVEF might be that patients enrolled in AMICA were generally too ill to benefit from AF ablation and sinus rhythm recovery. A direct comparison of the AMICA and CASTLE-AF studies' patient demographics showed that the AMICA study included sicker patients with more severe HF symptoms at baseline. AMICA study participants with ablation had lower LVEF (AMICA 27.6% vs. CASTLE-AF 32.5%) and more functional HF symptoms in NYHA Class III and IV (AMICA 60% vs. CASTLE-AF 31%). Thus, the AMICA study might indicate that not all AF and HF patients benefit from AF ablation despite sinus rhythm recovery. Patients with less advanced HF and better LVEF might gain more clinical benefits from sinus rhythm recovery. A subgroup analysis of the CASTLE-AF study supported this conclusion and showed that patients with NYHA III and LVEF <25% did not benefit from catheter ablation. CAMERA-MRI study (20) showed that loss of late gadolinium enhancement had significantly improved LVEF in the catheter ablation group (±19 ± 13% vs. 10 ± 11%) at 4.0 ± 0.9 years of follow-up. LVEF returned to normal in 19 patients (58%) compared with 4 patients (18%) in the advanced gadolinium-enhanced positive group (*P* = 0.008), suggesting that the use of CMR imaging can identify patients with ventricular scarring who are unlikely to respond well to catheter ablation. Catheter ablation showed an improvement in LVEF over 12 months, with milder symptoms of heart failure, a better level of baseline LVEF, and patients without ventricular fibrosis who were more likely to benefit from catheter ablation after resuming sinus rhythm.

The 6MWD test was a good assessment of overall mobility and functional reserve in patients with chronic heart failure. It was widely accepted that the ablation group achieved significant improvement in 6MWD (37). Our meta-analysis suggested that the 6MWD of patients in the catheter ablation therapy was 17.55 m improved than that in the drug therapy. Previous studies (37, 38) suggested that reduced stroke volume due to pathophysiological changes in HF and AF might be the basis for altered exercise tolerance. Therefore, a significant improvement in 6MWD could more effectively reflect the improvement of systolic cardiac function and rhythm recovery after successful catheter ablation. Similarly, the Minnesota Quality of Life Score (MLHFQ) was a valid measure of therapeutic efficacy, and its magnitude of improvement was similar to previous results associated with a favorable prognosis (39). It was particularly necessary to point out that catheter ablation not only improved symptoms early, but the CABANA HF sub-study (22) also confirmed that ablation patients experienced sustained improvements in quality of life over 5 years. These findings could be attributed to the restoration of a stable sinus rhythm, symptom relief, and improved health status following catheter ablation (40–42).

The reasons for the different re-hospitalization rates were contradictory among studies (19, 20, 22, 23, 25), and we believed that there might be different definitions of re-hospitalization. The AATAC study (25) referred to unplanned hospitalization as an arrhythmia-related cause or a complication of heart failure. Results of the AATAC study showed unplanned hospitalization rates (ablation 31% vs. drug 57%, *P < *0.001) had decreased by 45% (RR: 0.55, 95% CI: 0.39–0.76) at two years of follow-up. In the CASTLE-AF study (19), an HR of 0.56 (95% CI, 0.37–0.83; *P *= 0.004) was observed in the ablation group on reducing hospitalizations for worsening heart failure. These studies showed that the ablation group significantly reduced re-hospitalization rates when the cause was limited to worsening heart failure. However, the trend was less significant. So when the larger RAFT-AF study (23) measured the total number of hospitalizations, the statistical results were not statistically significant (RR: 1.03; 95% CI: 0.86–1.23, *P *= 0.733). In our meta-analysis, all hospitalizations mentioned in each study were considered, and the effect sizes of other small sample sizes were neutralized. Considering the more realistic clinical context, the ablation group did not increase the rate of compound re-hospitalization according to the analysis results.

It was easier to worry that catheter ablation might do harm to the patients. Early complications might include a catheter hole or excessive adjacent tissue ablation-related injuries. To our astonishment, this meta-analysis revealed that patients undergoing catheter ablation had no more adverse events than those in the drug group [OR: 1.06, (95% CI: 0.83–1.35), *P *= 0.66]. In the previous meta-analysis (34), the ablation groups had a higher rate of serious adverse events with no statistical difference [RR: 1.68 (95% CI: 0.58–4.85)]. Our results suggested that the adverse event rate of catheter ablation compared to drug treatment has been falling. The incidence of adverse events has decreased by nearly 20 years, mainly because catheter ablation technology is rapidly developing. For example, the iterative three-dimensional electrophysiological cardiac marking system made electrophysiological surgery more accurate and convenient. The emergence and improvement of adjustable bends, cold saline perfusion, and pressure monitoring catheters made electrophysiological examination and radiofrequency ablation more accurate. The emergence of a high-density mapping catheter further enabled an accurate judgment of the pathogenesis of atrial fibrillation. The development curve of technological progress was consistent with the downward trend of the adverse event rate. In particular, in the AMICA study (21), catheter ablation was relatively safe considering all patients' advanced heart failure and impaired left ventricular function. It also suggested that the current catheter ablation technology has become more suitable for AF patients with HF. 2019 AHA/ACC/HRS guideline recommended patients with symptomatic AF and HF with reduced left ventricular (LV) ejection fraction (HFrEF) to select catheter ablation for the sake of potentially lower mortality rate and hospitalization for HF (IIb) (43). 2021ESC guideline (44) recommends catheter ablation (IIa) for maintaining sinus rhythm with AF in patients with HF. AHA/ACC/HFSA guidelines for 2022 (45) recommended AF ablation for patients with symptomatic AF or HF to improve symptoms and quality of life (IIa). With the widespread application of cryoablation, pulsed field ablation (PFA) and other new technologies, the rate of adverse events would further decrease. We anticipate providing critical evidence for catheter ablation strategies in AF patients with HF. Additionally, future large-scale head-to-head comparative studies of PFA and radiofrequency ablation may give us a more accurate answer.

## Limitation

This study had several limitations. First, there was heterogeneity in the results of the statistics. In contrast, sensitivity analysis indicated that most meta-analysis results were stable. However, for re-hospitalization and changes in LVEF, AATAC and RAFT-AF studies should be excluded to make the meta-analysis results more stable. Second, since patients and doctors were not blind to treatment allocation, medical care and follow-up might differ after ablation, further affecting the results. Third, most studies only counted worsening heart failure as related to hospitalization rates, even though in the crowd of real people, re-hospitalization rates are also related to other factors. According to the study, catheter ablation did not increase re-hospitalization rates compared to drug treatment. However, given that the re-hospitalization rates in the study might have been underestimated, a more accurate combination re-hospitalization rate needs further study. Fourthly, although this study compared the efficacy difference between catheter ablation and drug therapy, it did not clearly distinguish between rhythm control and rate control. In order to answer this question, more high-quality RCTs were needed, even though current studies had not found a statistically significant difference in efficacy between the two drug strategies.

## Conclusion

With the development of ablation technology, the treatment of AF and HF modes has changed. Recent evidence suggests that for rhythm control in patients with AF and HF, catheter ablation improves left ventricular function, exercise tolerance, and quality of life and significantly reduces all-cause mortality and AF recurrence without increasing re-hospitalization or adverse events.

## Data Availability

The original contributions presented in the study are included in the article/**Supplementary Material**, further inquiries can be directed to the corresponding author/s.
