# Supplementary material for: Catheter ablation vs. drug therapy in the treatment of atrial fibrillation patients with heart failure: An update meta-analysis for randomized controlled trials
Source: Front Cardiovasc Med. 2023 Mar 8;10:1103567. doi: 10.3389/fcvm.2023.1103567 (PMC10031055; doi:10.3389/fcvm.2023.1103567)
Supplement: Supplementary file 1 [file Table1.docx]

| outcomes | beta1 | SE of beta1 | z | Prob > \|z\| |
| --- | --- | --- | --- | --- |
| all-cause mortality | -0.64 | 0.672 | -0.96 | 0.3389 |
| re-hospitalization | 2.94 | 1.174 | 2.51 | 0.0122 |
| change in LVEF | -3.02 | 10.222 | -0.30 | 0.7678 |
| AF recurrence | 2.81 | 0.642 | 4.37 | 0.0000 |
| QoL | 2.13 | 2.548 | 0.84 | 0.4030 |
| 6MWD | -5.70 | 3.630 | -1.57 | 0.1166 |
| adverse events | -0.11 | 0.661 | -0.17 | 0.8638 |

**Supplementary Table 1. Results of Regression-based Egger’s test for small-study effects.**

Random-effects model, Method: REML, H0: beta1 = 0; no small-study effects

LVEF = left ventricular ejection fraction; AF = atrial fibrillation; QoL = quality of life; 6MWD = six-minute walk distance.
