# Supplementary material for: Catheter ablation vs. drug therapy in the treatment of atrial fibrillation patients with heart failure: An update meta-analysis for randomized controlled trials
Source: Front Cardiovasc Med. 2023 Mar 8;10:1103567. doi: 10.3389/fcvm.2023.1103567 (PMC10031055; doi:10.3389/fcvm.2023.1103567)
Supplement: Supplementary file 2 [file Datasheet1.docx]

**(A).Heterogeneity analysis of all-cause mortality**


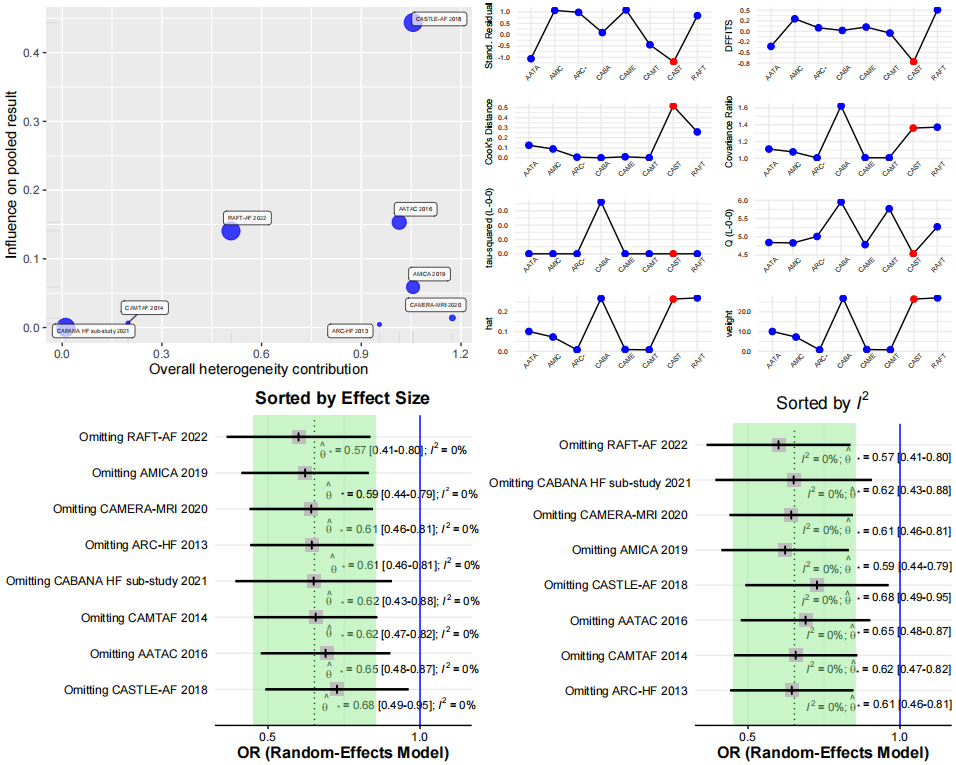


**(B).Heterogeneity analysis of re-hospitalization**


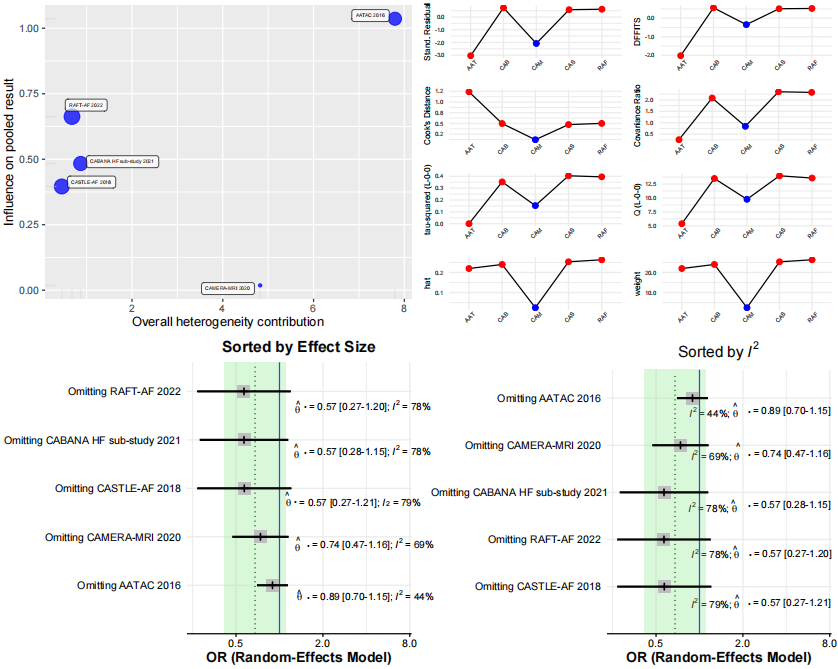


**(C).Heterogeneity analysis of change in LVEF**


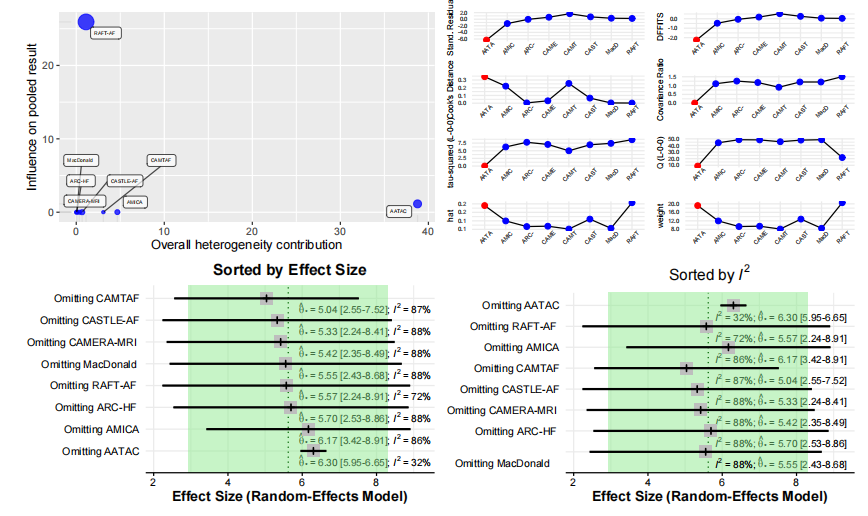


**(D).Heterogeneity analysis of AF recurrence**


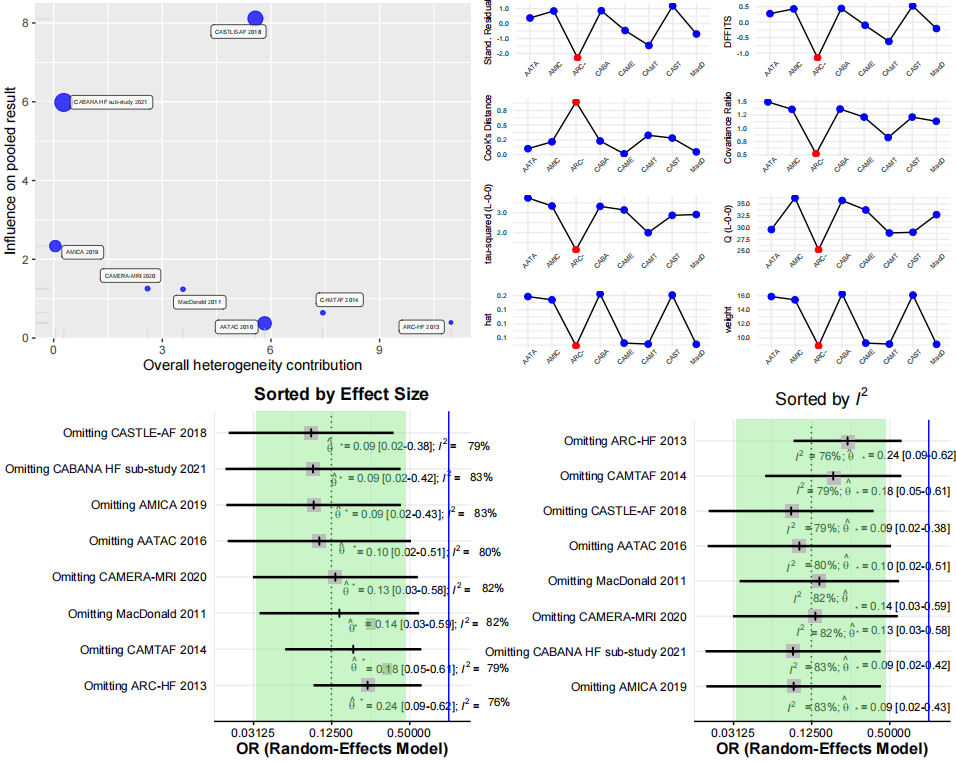


**(E).Heterogeneity analysis of quality of life**


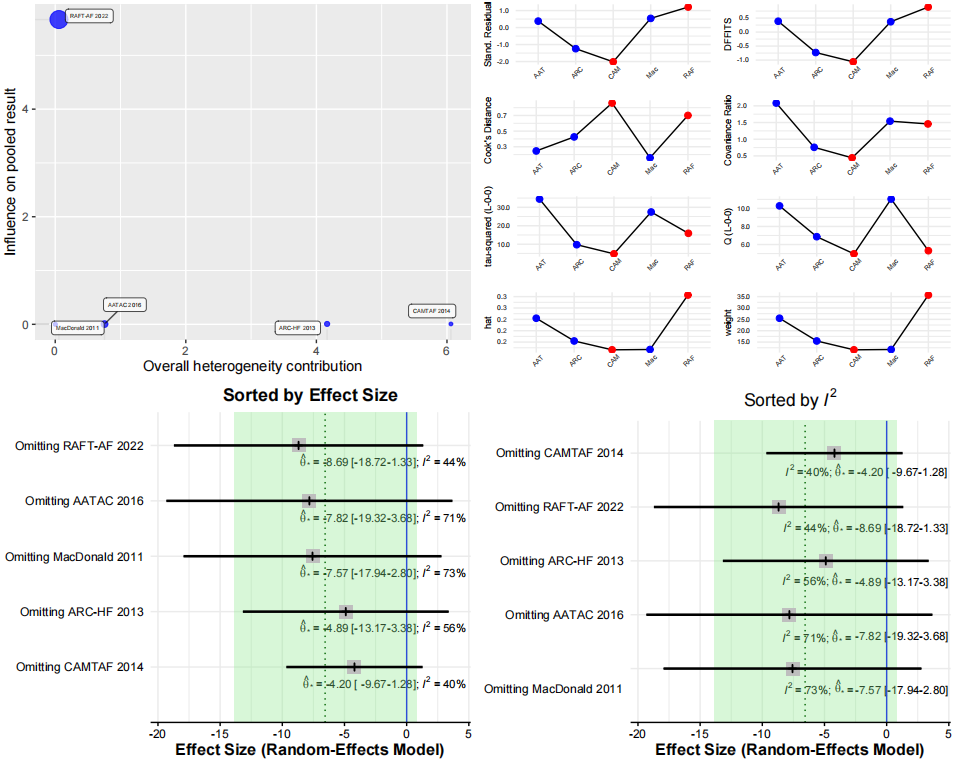


**(F).Heterogeneity analysis of 6-minute walk distance**


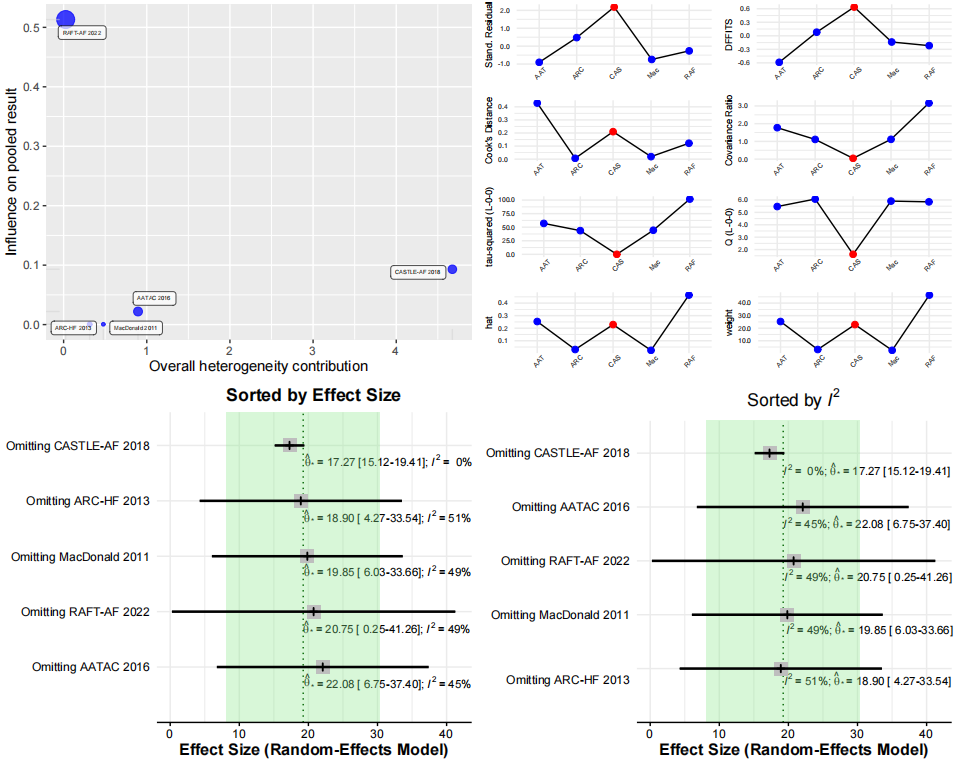


**(G).Heterogeneity analysis of adverse events**


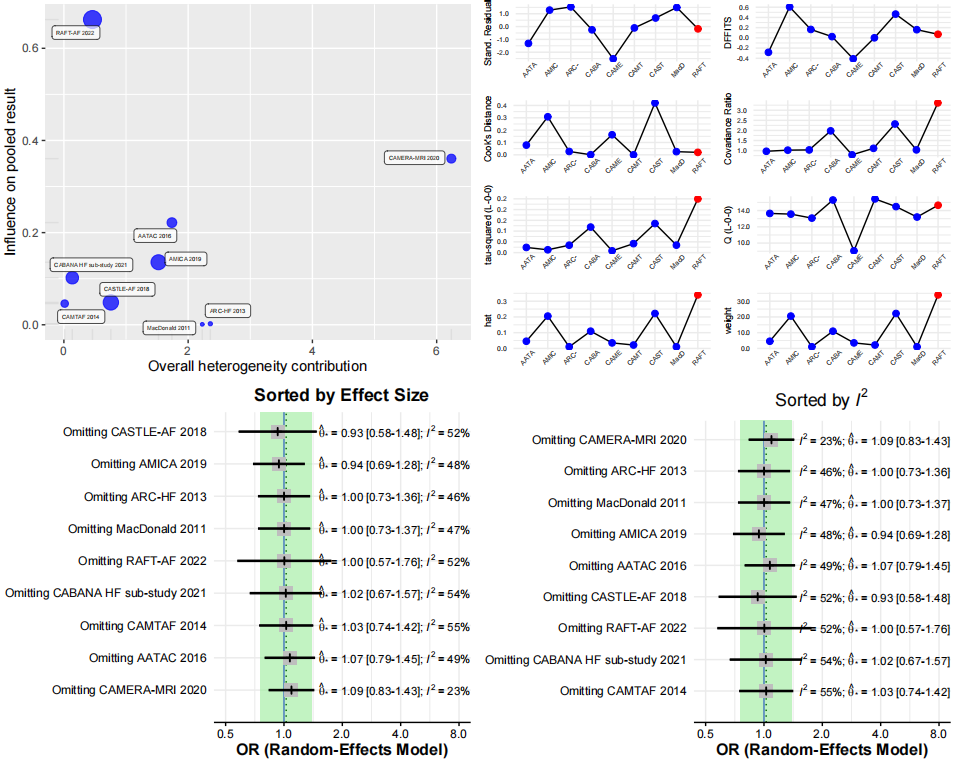


**Supplementary Figure 1. Results of the heterogeneity analysis**

ARC-HF = A Randomized Trial to Assess Catheter Ablation Versus Rate Control in the Management of Persistent Atrial Fibrillation in Heart Failure; CAMTAF = A Randomized Controlled Trial of Catheter Ablation Versus Medical Treatment of Atrial Fibrillation in Heart Failure; AATAC = Ablation vs. Amiodarone for Treatment of Persistent Atrial Fibrillation in Patients With Congestive Heart Failure and an Implanted Device; CAMERA-MRI = Catheter Ablation Versus Medication in Atrial Fibrillation and Systolic Dysfunction; CASTLE-AF = Catheter Ablation for Atrial Fibrillation with Heart Failure; AMICA = Catheter Ablation Versus Best Medical Therapy in Patients With Persistent Atrial Fibrillation and Congestive Heart Failure; CABANA HF sub-study = Ablation Versus Drug Therapy for Atrial Fibrillation in Heart Failure; RAFT-AF = Randomized Ablation-Based Rhythm-Control versus Rate-Control Trial in Patients with Heart Failure and Atrial Fibrillation.

Upper left corners are Baujat plots. In our meta-analyses, Baujat plots are used to detect studies that contribute excessively to heterogeneity. The horizontal axis shows each study's contribution to overall heterogeneity (measured by Cochran’s Q) , while the vertical axis shows its influence on the pooled effect size. We can consider the studies on the right side of the Baujat plots to be potentially relevant, since they contribute heavily to the overall heterogeneity. Baujat plots with studies in the upper right corners may be particularly influential since they affect both heterogeneity estimations and pooled effects.

Upper right corners are plots contains several influence diagnostics for each studies. The plot displays the value of different influence measures for each study. These measures are used to determine whether studies fit well into our meta-analysis model or not.

Lower left and right corners are forest plots for the overall effect and I^2^ heterogeneity of all meta-analyses with leave-one-out method.
