# Supplementary material for: Catheter ablation vs. drug therapy in the treatment of atrial fibrillation patients with heart failure: An update meta-analysis for randomized controlled trials
Source: Front Cardiovasc Med. 2023 Mar 8;10:1103567. doi: 10.3389/fcvm.2023.1103567 (PMC10031055; doi:10.3389/fcvm.2023.1103567)
Supplement: Supplementary file 3 [file Datasheet2.docx]

**(A).Re-hospitalization**

####
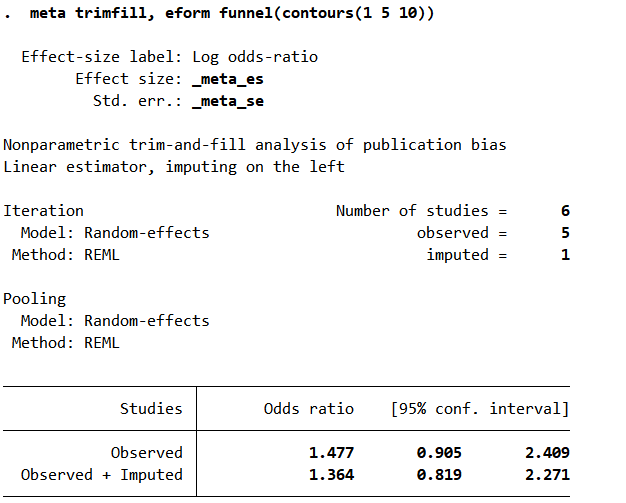

**(B).AF recurrence**


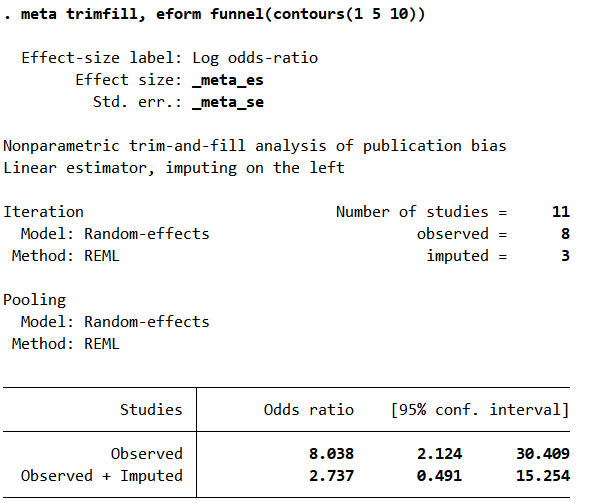

**Supplementary Figure 2. Trim-and-fill analysis of Re-hospitalization and AF recurrence**
