# Supplementary material for: Catheter ablation vs. drug therapy in the treatment of atrial fibrillation patients with heart failure: An update meta-analysis for randomized controlled trials
Source: Front Cardiovasc Med. 2023 Mar 8;10:1103567. doi: 10.3389/fcvm.2023.1103567 (PMC10031055; doi:10.3389/fcvm.2023.1103567)
Supplement: Supplementary file 4 [file Datasheet3.docx]

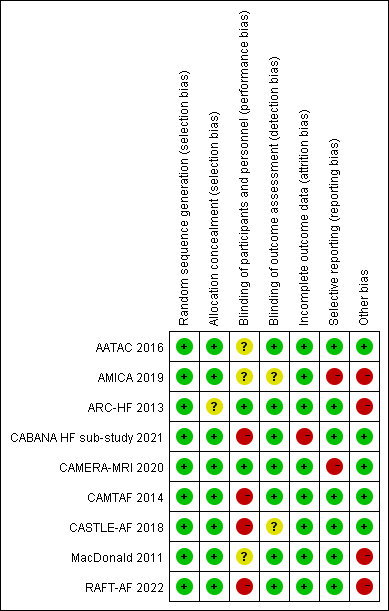


**Supplementary Figure 3. Risk of bias summary: review authors' judgements about each risk of bias item for each included study**. ARC-HF = A Randomized Trial to Assess Catheter Ablation Versus Rate Control in the Management of Persistent Atrial Fibrillation in Heart Failure; CAMTAF = A Randomized Controlled Trial of Catheter Ablation Versus Medical Treatment of Atrial Fibrillation in Heart Failure; AATAC = Ablation vs. Amiodarone for Treatment of Persistent Atrial Fibrillation in Patients With Congestive Heart Failure and an Implanted Device; CAMERA-MRI = Catheter Ablation Versus Medication in Atrial Fibrillation and Systolic Dysfunction; CASTLE-AF = Catheter Ablation for Atrial Fibrillation with Heart Failure; AMICA = Catheter Ablation Versus Best Medical Therapy in Patients With Persistent Atrial Fibrillation and Congestive Heart Failure; CABANA HF sub-study = Ablation Versus Drug Therapy for Atrial Fibrillation in Heart Failure; RAFT-AF = Randomized Ablation-Based Rhythm-Control versus Rate-Control Trial in Patients with Heart Failure and Atrial Fibrillation.
