# Supplementary material for: Catheter ablation vs. drug therapy in the treatment of atrial fibrillation patients with heart failure: An update meta-analysis for randomized controlled trials
Source: Front Cardiovasc Med. 2023 Mar 8;10:1103567. doi: 10.3389/fcvm.2023.1103567 (PMC10031055; doi:10.3389/fcvm.2023.1103567)
Supplement: Supplementary file 5 [file Datasheet4.docx]

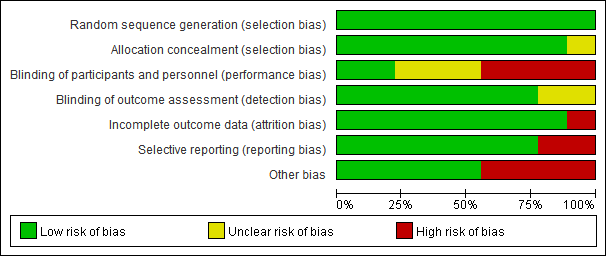


**Supplementary Figure 4. Risk of bias graph: review authors' judgements about each risk of bias item presented as percentages across all included studies.**
